# Inhibition of skin fibrosis via regulation of Th17/Treg imbalance in systemic sclerosis

**DOI:** 10.1038/s41598-025-85895-2

**Published:** 2025-01-09

**Authors:** Akiko Sekiguchi, Chikako Shimokawa, Tamotsu Kato, Akihiko Uchiyama, Yoko Yokoyama, Sachiko Ogino, Ryoko Torii, Hajime Hisaeda, Hiroshi Ohno, Sei-ichiro Motegi

**Affiliations:** 1 Department of Dermatology, Gunma University Graduate School of Medicine, 3-39-22, Showa, Maebashi, Gunma 371-8511 Japan; 2 Department of Parasitology, National Institute of Infectious Diseases, Shinjuku, Tokyo 162-8640 Japan; 3 Laboratory for Intestinal Ecosystem, RIKEN Center for Integrative Medical Sciences, Yokohama, Kanagawa 230-0045 Japan

**Keywords:** Systemic sclerosis, Th17/Treg balance, Fibrosis, Bleomycin, *Heligmosomoides polygilus*, Microbiota, Drug discovery, Immunology, Diseases, Medical research, Molecular medicine, Pathogenesis, Rheumatology, Risk factors, Signs and symptoms

## Abstract

Systemic sclerosis (SSc) is an idiopathic systemic connective tissue disorder characterized by fibrosis of the skin and internal organs, with growing interest in the imbalance between Th17 cells and regulatory T cells (Tregs) in the disease’s pathogenesis. *Heligmosomoides polygyrus* (Hp), a natural intestinal parasite of mice, is known to induce Tregs in the host. We aimed to investigate the effects of Hp-induced Tregs on bleomycin-induced dermal fibrosis and clarify the role of the Th17/Treg balance in SSc fibrosis. Infection with Hp suppressed the development of bleomycin-induced dermal fibrosis and the infiltration of CD3^+^ T cells and CD68^+^ macrophages. Flow cytometric analysis revealed that Hp infection increased Tregs and inhibited the induction of bleomycin-induced Th17 cells. Treg depletion nullified these effects, suggesting that Hp-induced Tregs may prevent bleomycin-induced dermal fibrosis and inflammation. Analysis of the intestinal microbiota showed that bacteria positively correlated with Tregs exhibited a negative correlation with Th17 cells and dermal fibrosis in mice. SSc patients with severe fibrosis displayed a distinct microbiota profile. These results suggest that alterations in the intestinal microbiota may contribute to the Th17/Treg imbalance in SSc and its progression. Enhancing Tregs to regulate the Th17/Treg imbalance may present a promising strategy for suppressing fibrosis in SSc.

## Introduction

Systemic sclerosis (SSc) is a diffuse connective tissue disorder characterized by the development of fibrosis in the skin and internal organs, immune abnormalities, and vascular disorders^1^. Despite advances in understanding its pathophysiology, the precise mechanisms underlying SSc remain unclear. It is widely accepted that T cell abnormalities serve as initiators of vascular damage and fibrosis in SSc. Recent studies have particularly highlighted the roles of Th17 cells and regulatory T (Treg) cells in this context^2,3^.

Th17 cells, which primarily differentiate in response to interleukin (IL)-6, are characterized by the expression of retinoic acid-related orphan receptor gamma t (RORγt) and the secretion of IL-17. These cells have been shown to be involved in the development of various autoimmune diseases^4,5^. In SSc, studies have demonstrated elevated frequencies of Th17 cells and cytokine levels in the peripheral blood, skin, and lungs of patients compared to healthy individuals. Additionally, these increases correlate with disease activity and excessive collagen production. Thus, Th17 cells are widely recognized as playing a crucial role in promoting inflammation, fibrosis, and autoimmunity in SSc^6,7^.

Treg cells, distinguished by the expression of forkhead box protein 3 (Foxp3) as a key transcription factor, play a critical role in maintaining immune self-tolerance and modulating both autoimmune and protective immune responses. Treg cells exert regulatory functions by actively suppressing effector T cells and antigen-presenting cells through various mechanisms. These include the secretion of immunosuppressive cytokines like TGF-β and IL-10, the generation of metabolic adenosine, and cell-cell interactions via surface molecules such as cytotoxic T lymphocyte-associated antigen 4 (CTLA-4)^8^. Despite the well-established role of Treg cells in immune regulation, studies on their frequency in SSc have yielded conflicting findings, with some indicating higher levels and others suggesting lower levels compared to healthy individuals^9,10^. However, most studies agree that regardless of their frequency, Treg cells in SSc exhibit compromised suppressive function^11^. Recent research has emphasized the importance of the Th17/Treg balance rather than individual T cell subsets in autoimmune diseases^12,13^. Nevertheless, research specifically addressing this imbalance in SSc remains limited, and a detailed understanding of its mechanisms and clinical significance is still lacking.

Helminths are multicellular parasites of humans and other animals, renowned for their sophisticated ability to modulate their hosts’ immune responses^14^. Typically, anti-parasite immunity relies on a robust Th2-type immune response^15^. However, helminths have evolved various mechanisms to either divert or suppress this response, thereby ensuring their survival^14,16^. Notably, immune responses induced by helminths include the differentiation and expansion of host Treg cells, which can significantly impact inflammatory and autoimmune conditions. Epidemiological studies have demonstrated that the incidence of helminth infections is negatively correlated with the incidence of immune-mediated disorders on a global scale^17^. Additionally, infection with the natural intestinal helminth *Heligmosomoides polygyrus* (Hp) in mice has been shown to increase Treg cell populations and alleviate symptoms in various animal models of allergic and autoimmune diseases^18,19^. Such immune modulation by helminths may occur through both direct effects on the host immune system and indirect effects mediated by the intestinal microbiota^16^. The intestinal microbiota, along with its enzymatic by-products, significantly influences host physiological functions such as metabolism and immune responses.

These findings suggest that helminth infection could serve as a valuable model for investigating the role of Treg cells in immune-mediated disorders. This study aims to examine the effects of helminth-induced Treg cell expansion on the development of bleomycin-induced dermal fibrosis and to elucidate the role of the Th17/Treg imbalance in the pathogenesis of fibrosis in SSc, as well as its potential as a target for new therapeutic strategies.

## Results

### Helminth infection suppressed the bleomycin-induced dermal fibrosis and inflammation in mice

**Fig. 1:**
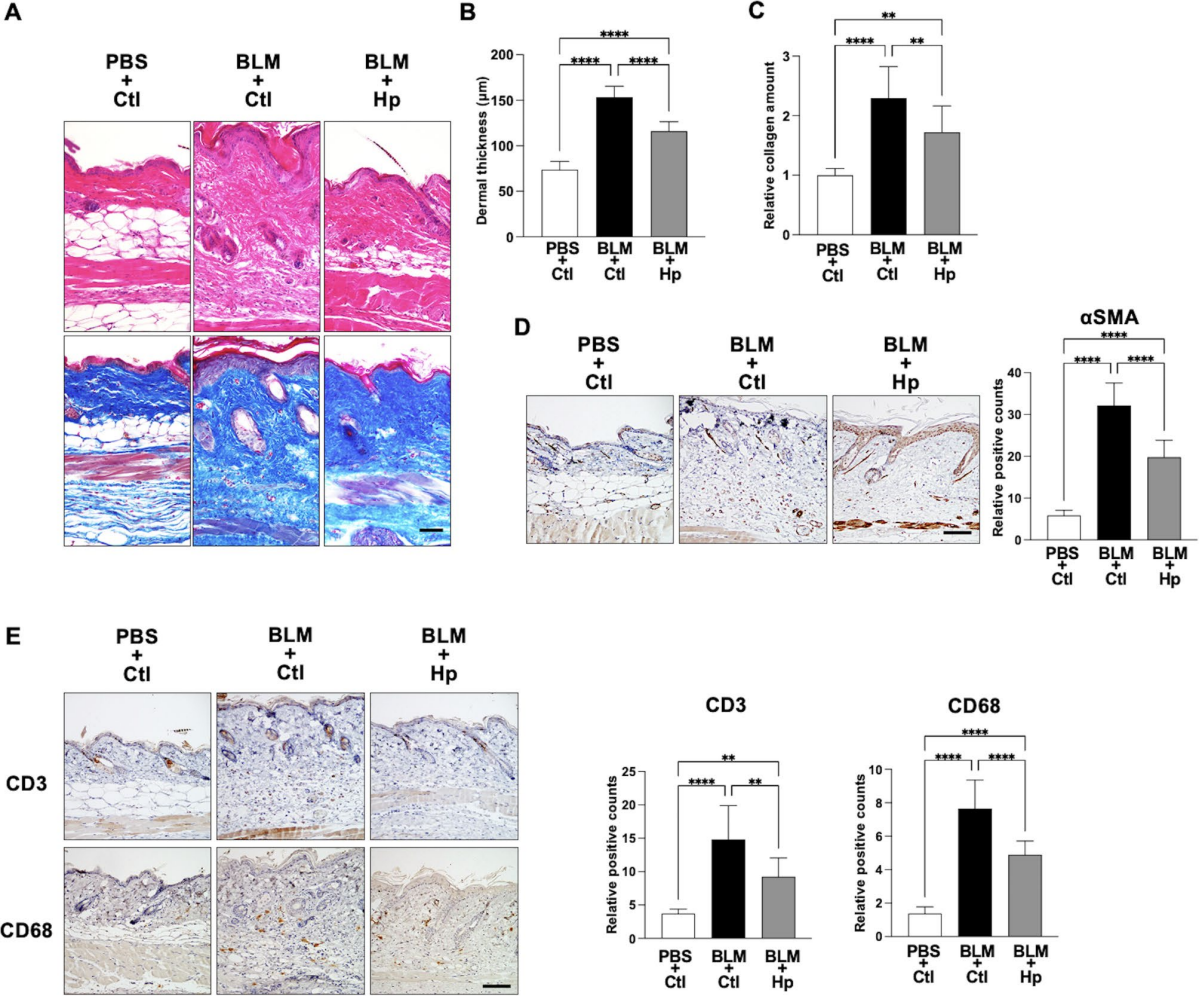
Helminth infection suppressed the bleomycin-induced dermal fibrosis and inflammation in mice. (**A**) Representative images of H&E staining (top) or Masson trichrome staining (bottom) of skin sections taken 14 days after initiation of subcutaneous injection of bleomycin or control PBS into Hp-infected or uninfected mice. Scale bar = 50 μm. (**B**) Quantification of dermal thickness of the lesional skin in mice. Values were determined in 6–8 random microscopic fields in *n* = 9 mice per groups. (**C**) Relative amounts of soluble collagen in 5 mm diameter skin sections. *n* = 10 mice per groups. (**D**) Representative immunochemical staining images of skin sections to show the numbers of αSMA^+^ myofibroblasts. Right panel shows quantification of positive cells in 6 random microscopic fields in the dermis. *n* = 7–10 mice per groups. Scale bar = 50 μm. (**E**) Representative immunochemical staining images of skin sections taken 5 days after the initiation of subcutaneous injection of bleomycin or control PBS into Hp-infected or uninfected mice to show the numbers of CD3^+^ T cells (top) and CD68^+^ macrophages (bottom). Right panels show quantification of each positive cells in 6 random microscopic fields in the dermis. *n* = 8–10 mice per groups. Scale bar = 50 μm. Values represent mean ± SEM. **P < 0.01 and ****P < 0.0001. One-way ANOVA followed by Tukey-Kramer test was used for (**B**–**E**). Hp, *Heligmosomoides polygilus*; BLM, bleomycin; Ctl, control.

To investigate the effect of helminth infection on skin fibrosis, we compared the development of bleomycin-induced dermal fibrosis in mice with and without prior Hp infection. Fourteen days after the initiation of bleomycin injection, H&E staining revealed that the development of dermal thickness induced by bleomycin was significantly suppressed in mice pre-infected with Hp compared to those that were not infected (Fig. 1A, B). Masson trichrome staining confirmed that the fibrosis induced by bleomycin was suppressed in mice pre-infected with Hp compared to uninfected controls (Fig. 1A). Additionally, the increase in skin collagen levels caused by bleomycin was significantly less in Hp-infected mice compared to non-infected mice (Fig. 1C). Immunohistochemical staining showed that the number of αSMA^+^ myofibroblasts induced in Hp-infected mice by bleomycin injections was significantly lower compared to uninfected controls (Fig. 1D). Bleomycin-treated mice are known to mimic the inflammatory aspects of SSc patients, which may be associated with the induction of fibrosis^20,21^. Therefore, we next examined the effect of Hp infection on bleomycin-induced inflammation. Five days after the initiation of bleomycin injection, immunohistochemical staining showed that the infiltration of CD3^+^ T cells and CD68^+^ macrophages in the skin of Hp-infected mice was significantly lower compared to uninfected controls (Fig. 1E).

### CD4^+^ Treg cells are required for the suppressive effect of helminth infection on bleomycin-induced dermal fibrosis and inflammation

**Fig. 2:**
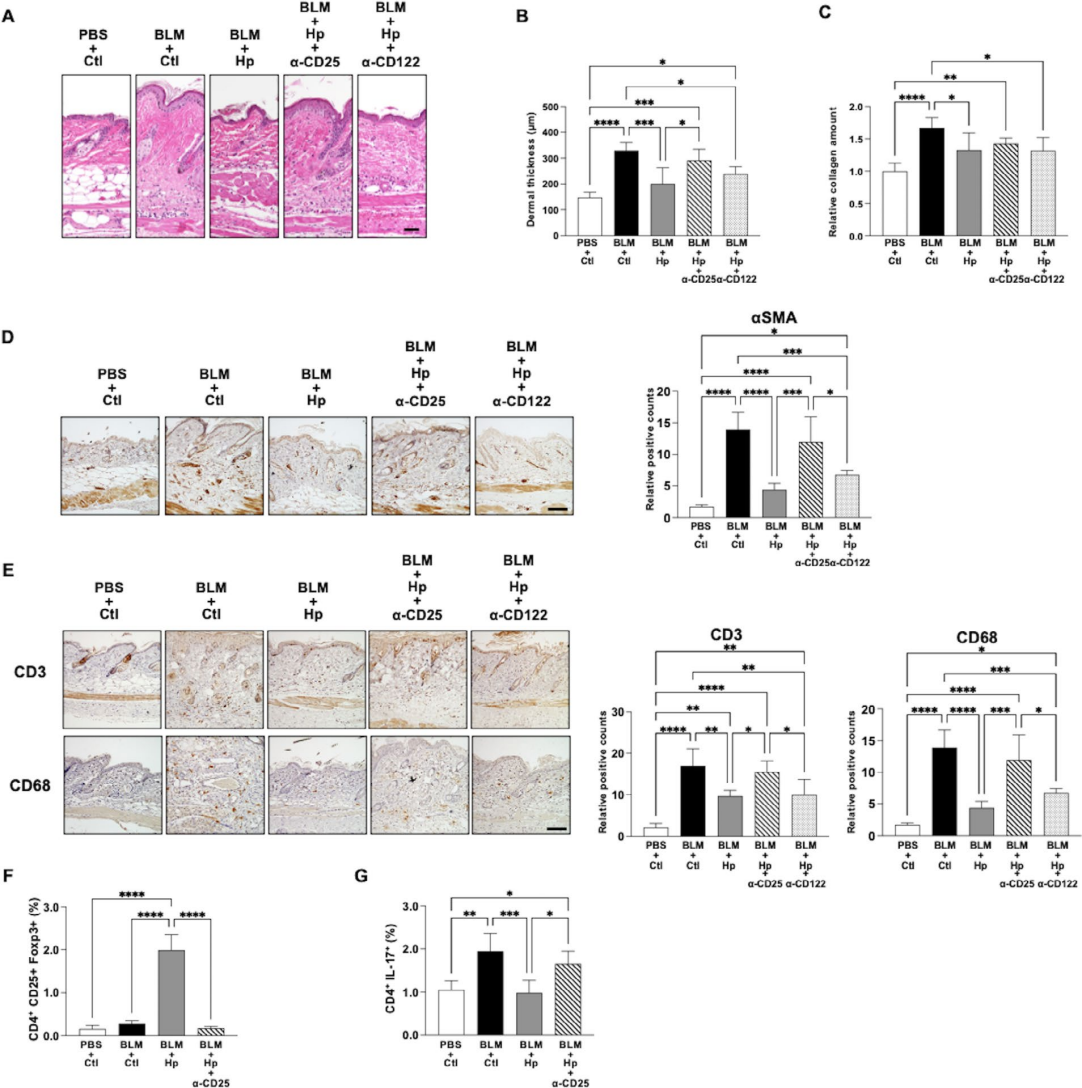
CD4^+^ Treg cells are required for the suppressive effect of helminth infection on bleomycin-induced dermal fibrosis and for regulating the Th17/Treg imbalance. (**A**) Representative images of H&E staining of skin sections taken 14 days after the initiation of subcutaneous injection of bleomycin or control PBS into Hp-infected or uninfected mice injected intraperitoneally with anti-CD25 antibody, anti-CD122 antibody or control PBS. Scale bar = 50 μm. (**B**) Quantification of dermal thickness of the lesional skin in mice. Values were determined in 6 random microscopic fields in *n* = 5 mice per groups. (**C**) Relative amounts of soluble collagen in 5 mm diameter skin sections. *n* = 10 mice per groups. (**D**) Representative immunochemical staining images of skin sections to show the numbers of αSMA^+^ myofibroblasts. Right panel shows quantification of positive cells in 6 random microscopic fields in the dermis. *n* = 4–5 mice per groups. Scale bar = 50 μm. (**E**) Representative immunochemical staining images of skin sections taken 5 days after the initiation of subcutaneous injection of bleomycin or control PBS into Hp-infected or uninfected mice injected intraperitoneally with anti-CD25 antibody, anti-CD122 antibody or control PBS to show the numbers of CD3^+^ T cells (top) and CD68^+^ macrophages (bottom). Right panels show quantification of each positive cells in 6 random microscopic fields in the dermis. *n* = 5 mice per groups. Scale bar = 50 μm. (**F**,**G**) Quantification by flow cytometry of Treg cells defined as CD25^+^Foxp3^+^ cells (**F**) and Th17 cells defined as Th17^+^ cells (**G**) among gated CD4^+^ T cells in the inguinal lymph node taken 14 days after the initiation of subcutaneous injection of bleomycin or control PBS into Hp-infected or uninfected mice injected intraperitoneally with anti-CD25 antibody, anti-CD122 antibody or control PBS. Values represent mean ± SEM. *P < 0.05, **P < 0.01, ***P < 0.001 and ****P < 0.0001. One-way ANOVA followed by Tukey-Kramer test was used for (**B**–**G**). Hp, *Heligmosomoides polygilus*; BLM, bleomycin; Ctl, control.

Next, to clarify the role of Treg cells in mediating the suppressive effect of Hp infection on the development of bleomycin-induced fibrosis and inflammation, we examined the effects of Treg cell depletion. Although Treg cells are typically characterized as CD3^+^CD4^+^CD25^+^Foxp3^+^ T cells, a subset of CD8^+^ Treg cells (CD3^+^CD8^+^CD44^+^CD122^+^Ly49^+^) has also been identified in mice and humans and has been reported to be associated with autoimmune diseases^19,22^. To deplete CD25^−^expressing cells, which include CD4^+^ Treg cells, and CD122-expressing cells, which include CD8^+^ Treg cells, we administered anti-CD25 antibody or anti-CD122 antibody intraperitoneally, respectively. Fourteen days after the initiation of bleomycin injection, the suppressive effect of Hp infection on the development of bleomycin-induced fibrosis was not observed in mice treated with anti-CD25 antibody, whereas it was maintained in mice treated with anti-CD122 antibody (Fig. 2A–C). The suppressive effect of Hp infection on the infiltration of αSMA^+^ myofibroblasts induced by bleomycin injections was not observed in mice treated with anti-CD25 antibody, whereas it was maintained in mice treated with anti-CD122 antibody (Fig. 2D). Five days after the initiation of bleomycin injection, immunohistochemical staining revealed that the suppressive effect of Hp infection on the infiltration of CD3^+^ T cells and CD68^+^ macrophages induced by bleomycin was not observed in mice treated with anti-CD25 antibody, whereas it was maintained in mice treated with anti-CD122 antibody (Fig. 2E). These results suggest that Treg cells, specifically CD4^+^ Treg cells, are required for the suppressive effect of Hp infection on the development of bleomycin-induced dermal fibrosis and inflammation.

### CD4^+^ Treg cells induced by helminth infection regulate the Th17/Treg imbalance in a bleomycin-induced SSc mouse model

We next examined the effect of Hp infection on the balance between Th17 and CD4^+^ Treg cells in the bleomycin-induced SSc mouse model. First, we confirmed that bleomycin injections increased the number of Th17 cells, as assessed by flow cytometric analysis of mouse inguinal lymph nodes. CD4^+^CD25^+^Foxp3^+^ Treg cells were significantly elevated in the Hp-infected bleomycin-induced SSc model mice compared to the uninfected controls (Fig. 2F). Importantly, the number of CD4^+^CD17^+^ Th17 cells induced by bleomycin was significantly lower in the Hp-infected mice compared to the uninfected controls (Fig. 2G). Notably, this suppressive effect of Hp infection on the enhancement of Th17 cells induced by bleomycin was not observed in mice depleted of Treg cells by anti-CD25 antibody treatment. These findings suggest that Hp infection may suppress the development of bleomycin-induced Th17 cells through the induction of Treg cells.

## Association of intestinal microbiota in bleomycin-induced dermal fibrosis and Th17/Treg balance in SSc mouse model

**Fig. 3:**
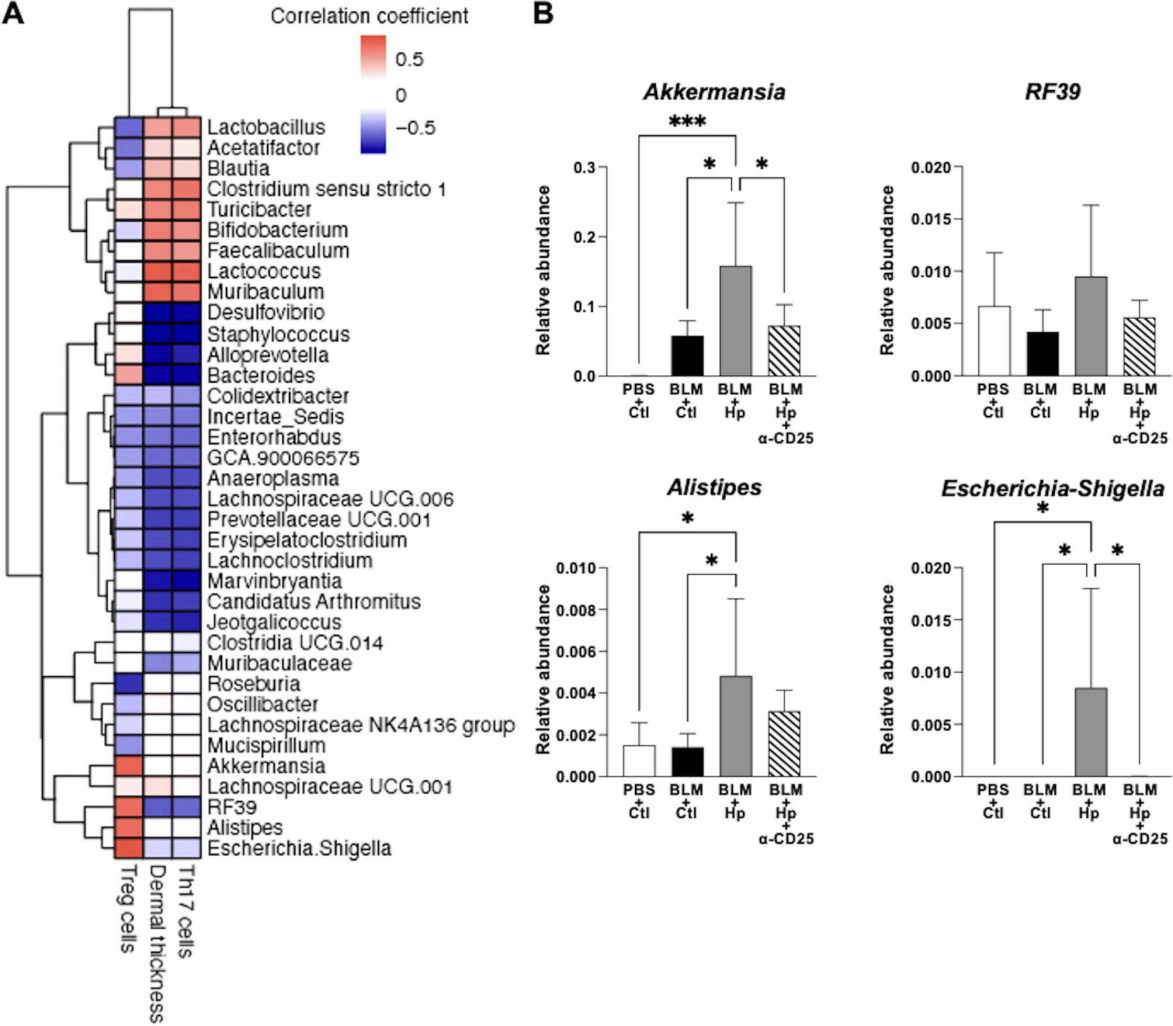
Association of intestinal microbiota in bleomycin-induced dermal fibrosis and Th17/Treg imbalance in SSc mouse model. (**A**,**B**) Investigations of bacterial genera in faeces taken 14 days after the initiation of subcutaneous injection of bleomycin or control PBS into Hp-infected or uninfected mice injected intraperitoneally with anti-CD25 antibody or control PBS. (**A**) Heatmap showing the strength of correlation based on Pearson correlation coefficient on a color scale between abundance of faecal bacterial genera and the number of Treg cells, dermal thickness, and the number of Th17 cells, respectively. (**B**) Relative abundance of bacteria in mouse faeces that were positively correlated with the number of Treg cells. *n* = 5–6 mice per group. Values represent mean ± SEM. *P < 0.05, ***P < 0.001. One-way ANOVA followed by Tukey-Kramer test was used for (**B**). Hp, *Heligmosomoides polygilus*; BLM, bleomycin; Ctl, control; Treg, regulatory T.

To identify the mechanisms underlying the regulatory effect of Hp infection on bleomycin-induced dermal fibrosis and Th17/Treg imbalance, we investigated the alterations in the intestinal microbiota. We focused on three key parameters: the number of Treg cells, dermal thickness, and the number of Th17 cells, analyzing their correlations with the faecal microbiota of mice. The pattern of bacterial abundance was similar between dermal thickness and the number of Th17 cells (Fig. 3A). The number of Treg cells in mice demonstrated a strong positive correlation with the abundance of *Akkermansia*, *RF39*, *Alistipes*, and *Escherichia-Shigella*. Conversely, dermal thickness and the number of Th17 cells showed rather a negative correlation with these bacteria (Fig. 3A). We also observed that the abundance of these bacteria increased in Hp-infected mice, but not in those receiving anti-CD25 antibody (Fig. 3B). These results suggest that these bacteria could potentially contribute to the regulatory effects of Hp infection on the bleomycin-induced dermal fibrosis and Th17/Treg imbalance, possibly through the modulation of Treg cells.

### Analysis of characteristics of microbiota in patients with SSc and comparison with clinical symptoms

**Fig. 4:**
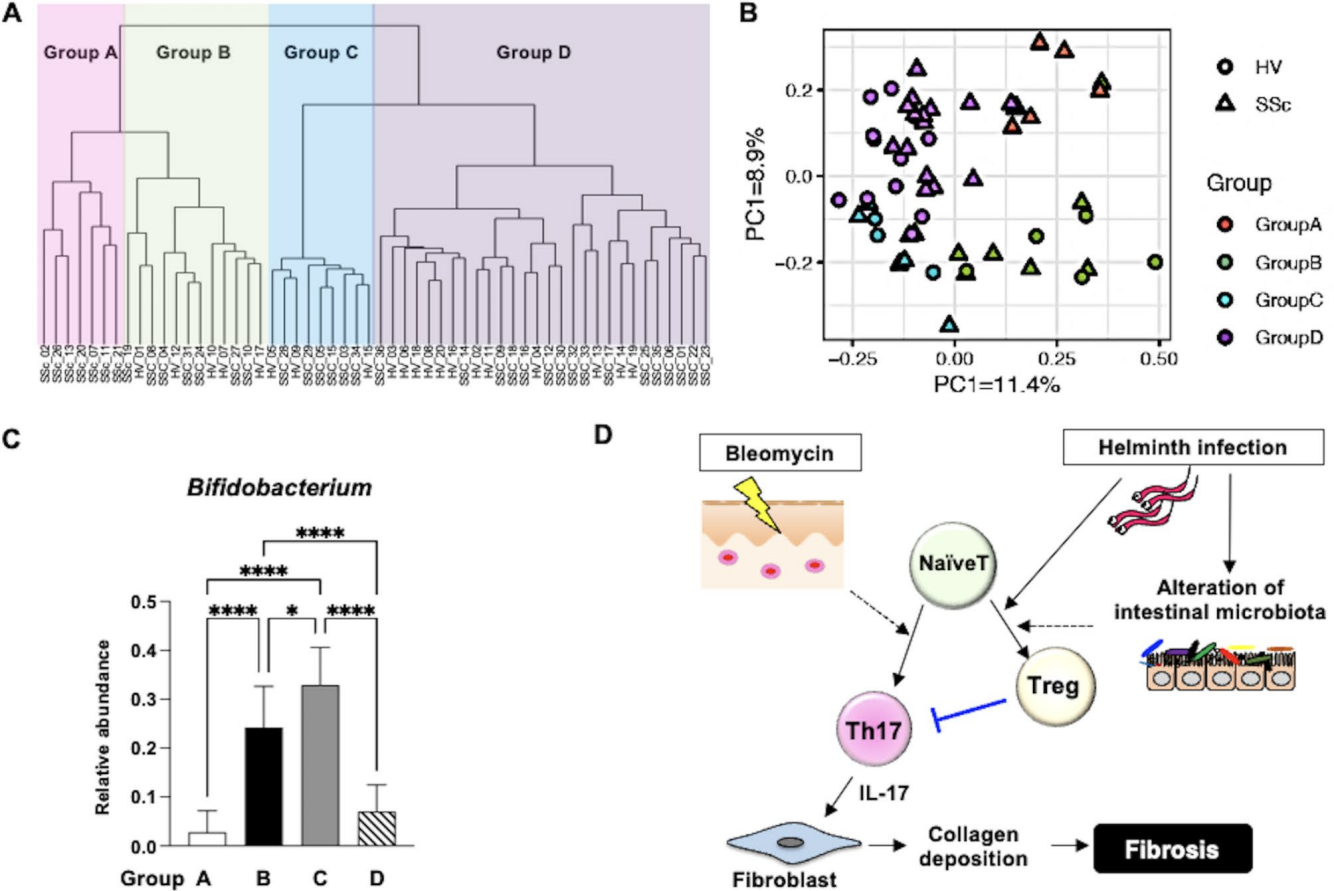
Characteristics of the microbiota in patients with SSc. (**A**,**B**) The faecal bacterial composition of 36 SSc patients and 20 healthy individuals is shown in hierarchical clustering trees (**A**) and Principal Coordinate Analysis (PCoA) plots (**B**), based on Bray-Curtis distances calculated from the OTU distribution across samples. (**C**) Relative abundance of *Bifidobacterium* characterized by a decrease in the intestinal microbiota of SSc patients in group A. (**D**) Model of inhibition of bleomycin-induced dermal fibrosis by helminth infection-mediated regulation of Th17/Treg balance. Values represent mean ± SEM. One-way ANOVA followed by Tukey-Kramer test was used for (**C**). *P < 0.05 and ****P < 0.0001. SSc, systemic sclerosis; PCoA, principal coordinates analysis; OTU, operational taxonomic unit; Treg, regulatory T; HV, healthy volunteer.

To determine whether patients with SSc have a unique intestinal microbiota pattern that may contribute to the pathogenesis of the disease, we examined the faecal microbiota of 36 SSc patients and compared it to that of 20 healthy individuals. Hierarchical clustering divided the participants into four groups according to branch distance thresholds (Fig. 4A). Group A consisted of only SSc patients and and formed an independent cluster in the upper right of the Principal Coordinate Analysis (PCoA) plot (Fig. 4B). These results suggest that SSc patients can be categorized into two groups based on their microbiota characteristics.

To characterize the SSc patients in group A, we examined their clinical characteristics and compared them to those of patients not included in Group A (Table 1). Among the 36 SSc patients, 7 belonged to group A. We found that the frequency of diffuse cutaneous SSc (dcSSc) was significantly higher in patients in group A than in those in the other groups (85.7% vs. 44.8%, *P* < 0.05). In addition, the total skin score was significantly higher in patients in group A than those in the other groups (19.1 ± 3.1 vs. 7.5 ± 0.9, *P* < 0.01). Regarding complications, digital ulcers (DUs) and interstitial lung disease (ILD) occurred more frequently in patients in group A than those in the other groups (DU 71.4% vs. 27.6% (*P* < 0.05), ILD 85.7% vs. 41.4% (*P* < 0.05)). These results suggest that SSc patients with characteristics such as severe skin sclerosis, ILD, and DUs may have a unique microbiota that differs from that of other SSc patients and healthy individuals. We also examined the bacteria characterizing group A and found that SSc patients in group A had lower levels of *Bifidobacterium* compared to patients in the other groups (Fig. 4C).

**Table 1:** Comparison of demographic and clinical characteristics of SSc patients included or not included in group A.

	SSc patients (*n* = 36)		*P*-value
	Group A (19.4%: *n* = 7)	Non-Group A (80.6%: *n* = 29)	
Male patients	57.1% (4/7)	20.7% (6/29)	0.05
Disease duration (years; mean ± SD)	5.6 ± 2.4	5.5 ± 4.3	0.96
dcSSc	85.7% (6/7)	44.8% (13/29)	< 0.05*
Total skin score (mean ± SE)	19.1 ± 3.1	7.5 ± 0.9	< 0.01**
Complications			
Digital ulcer	71.4% (5/7)	27.6% (8/29)	< 0.05*
Interstitial lung disease	85.7% (6/7)	41.4% (12/29)	< 0.05*
Pulmonary artery hypertension	0% (0/7)	10.3% (3/29)	0.37
GERD	71.4% (5/7)	58.6% (17/29)	0.53
Autoantibodies			
ACA	28.6% (2/7)	51.7% (15/29)	0.27
Topo I	57.1% (4/7)	24.1% (7/29)	0.09
RNP	0% (0/7)	6.9% (2/29)	0.48
RNAP	0% (0/7)	13.8% (4/29)	0.3

*SD* standard deviation, *SE* standard error, *SSc* systemic sclerosis, *dcSSc* diffuse cutaneous SSc, *GERD* gastroesophageal reflux disease, *ACA* anticentromere antibody, *Topo I* anti-topoisomerase I antibody, *RNP* anti‐U1 RNP antibody, *RNAP* anti‐RNA polymerase III antibody.

*P*-values were calculated using Student’s *t*-test (two-tailed) or Chi-square analysis. *P < 0.05 and **P < 0.01.

## Discussion

Increasing evidence suggests that Th17 cells play a crucial role in the development of fibrosis in SSc by initiating cascades of proliferative and inflammatory cytokines. In vitro studies have demonstrated that IL-17 enhances the expression of key fibrotic mediators such as TGF-β and CTGF in murine fibroblasts, leading to increased collagen synthesis in these cells^2^. IL-17 produced by Th17 cells in active SSc patients has been shown to induce fibroblast proliferation as well as collagen production and secretion^23^. Research using mouse models has revealed that IL-17 A knockout mice exhibit improvements in skin fibrosis in two fibrotic animal models (bleomycin-induced and tight skin (TSK)). Moreover, the expression of TGF-β and CTGF in the skin was inhibited in bleomycin-treated IL-17 A knockout mice^2^. Th17 cells in SSc also affect endothelial cells. IL-17 A derived from the serum of SSc patients has been shown to induce the expression of intercellular adhesion molecule 1 (ICAM-1) and vascular cell adhesion molecule 1 (VCAM-1), as well as C-C motif chemokine ligand 20 (CCL-20) and C-X-C motif chemokine receptor 4 (CXCR-4) in endothelial cells, leading to endothelial inflammation^6,24^. Endothelial cell injury is associated with fibrin deposition, leading to narrowing of the vascular lumen and vessel occlusion, which contributes to microangiopathy. Extensive microangiopathy results in chronic tissue hypoxia, thereby exacerbating tissue fibrosis. Additionally, the migration of leukocytes and inflammatory cytokines from the vasculature to the extracellular matrix (ECM) aggravates ECM fibrosis. Thus, IL-17 A plays a central role in both inflammation and fibrosis observed in SSc. Indeed, the IL-17 A receptor antagonist brodalumab has been shown to suppress fibrosis in SSc patients, representing a promising therapeutic option^25^.

Up-to-date evidence indicates that that Th17 and Treg cells regulate autoimmune responses through their interactions^26–28^. Generally, antigen-activated naive T cells express both RORγt and Foxp3 in the presence of TGF-β. Foxp3 suppresses the transcriptional activation of RORγt and promotes Treg cell proliferation, thereby preventing the abnormal activation of autoimmunity^27,28^. However, in the presence of inflammatory cytokines such as IL-6, IL-1, and IL-21, naive CD4^+^ T cells are induced to differentiate into Th17 cells and inhibited from differentiating into Treg cells^26,27^. Furthermore, studies have demonstrated that cytokine environments involving IL-6 may promote the differentiation of certain regulatory T cells into cells that produce IL-17, akin to Th17 cells^29,30^. Given that IL-6 plays a crucial role in the pathogenesis of SSc and is elevated in the blood and tissues of patients in the early stages of the disease^31^, it is expected that the Th17/Treg balance in SSc is skewed toward the Th17 subset, contributing to extensive fibrosis and persistent inflammation^26,32^. In this study, we investigated the role of Th17/Treg cell balance in the pathogenesis of fibrosis in SSc using helminth-induced Treg cells. Our results showed that in a mouse model of fibrosis, there is an imbalance in the Th17/Treg skewed towards Th17. Moreover, an increase in Treg cells can help regulate this imbalance and inhibit the progression of fibrosis. These findings provide evidence that regulating the Th17/Treg imbalance through increasing Treg cells could represent a novel and promising therapeutic strategy for fibrosis in SSc.

Intestinal bacteria produce a variety of metabolites in the process of symbiosis with the host, which also affect the immune system as molecules that communicate with host cells. Short-chain fatty acids (SCFAs) are the main metabolites produced by the fermentation of dietary fiber and resistant starch performed by certain bacteria, including acetic acid, propionic acid, and butyric acid^33^. SCFAs are known to promote the differentiation and migration of Treg cells^34,35^ and have been reported to have anti-inflammatory effects against various autoimmune diseases such as systemic lupus erythematosus (SLE), rheumatoid arthritis (RA), and multiple sclerosis (MS)^36–38^. The genus *Akkermansia*, *RF39* and *Alistipes*, which were shown to be positively correlated with the number of Treg cells in our investigation of the intestinal microbiota in helminth-infected SSc model mice, have been reported to affect host immunity through the production of SCFAs^39–41^. Considering the negative correlation between these bacteria and the number of Th17 cells and dermal thickness, it is suggested that the regulatory effects of helminth infection via Treg induction on bleomycin-induced Th17/Treg imbalance and dermal fibrosis may be mediated by the SCFAs produced by these bacteria. In addition, depletion of Treg cells reduced the abundance of bacteria, which was increased by Hp infection. It is possible that Treg depletion impairs the regulation of the intestinal immune system, resulting in an excessive immune response and altering the composition of the intestinal microbiota. Another mechanism by which helminth infection induces host Treg responses involves the secretion of metabolites and immune-regulatory factors by the parasites themselves. For example, Hp secretes hundreds of proteins in its excretory/secretory products, some of which have been shown to induce Treg cell differentiation from naive CD4^+^ T cells in vitro^42^. Further analysis has identified an active protein, *H. polygyrus* TGF-β mimic (Hp-TGM), which mimics the function of TGF-β by binding to the TGF-β receptor and inducing Smad signaling^43^. These immune-regulatory factors also interact with bystander cells such as dendritic cells and macrophages and help induce Treg cells^44^. It is possible that these factors contribute to Treg induction in conjunction with changes to the intestinal microbiota. Further studies are needed to investigate these mechanisms more comprehensively.

Although we analyzed the intestinal microbiota of SSc patients with the expectation that bacteria showing a strong positive correlation with Treg cells in our mouse model would be reduced in SSc patients, we found no significant association between these bacteria and SSc. However, interestingly, patients with severe fibrosis demonstrated a distinct microbiota pattern compared to those with mild disease and healthy controls, which supports the association between fibrosis and microbiota in SSc. Our study did not determine whether this alteration in microbiota is a trigger for SSc, contributes to disease progression, or is simply a result of the disease. Nonetheless, it provides evidence that regulating immunity via the intestinal microbiota could have potential clinical applications for SSc fibrosis. For instance, we observed a reduction in *Bifidobacterium*, a bacterium that modulates host immunity by producing SCFAs and inducing Treg cells. Addressing this dysbiosis and restoring a more balanced microbiota may potentially help in treating SSc fibrosis. Further research is needed to explore this potential.

There are several limitations to this study. First, analysis of Th17/Treg balance involved in the pathogenesis of SSc fibrosis is not sufficient to examine lymph nodes alone. There is a possibility of differing outcomes between lymphoid and fibrotic tissues. Second, in this study, several candidate bacteria that may be associated with the regulatory effect of Hp infection on bleomycin-induced Th17/Treg imbalance and dermal fibrosis have been identified, but whether these bacteria are involved has not been verified. To directly assess the role of specific bacteria in regulating immune function and fibrosis, future studies could involve the use of germ-free mice, fecal microbiota transplantation, or selective bacterial depletion. Third, the association between intestinal microbiota and Th17/Treg imbalance in SSc patients was not examined in this study and it remains an intriguing prospect for future research.

Finally, we propose a mechanistic model for suppressing dermal fibrosis in SSc by regulating the Th17/Treg imbalance through the induction of Treg cells (Fig. 4D). Bleomycin induces Th17 cells and shifts the Th17/Treg balance toward Th17, mimicking the Th17/Treg imbalance observed in SSc. Th17 cells produce IL-17, which activates fibroblasts, leading to collagen production and fibrosis. Helminth infection inhibits the development of bleomycin-induced fibrosis by promoting the differentiation of naive T cells into Treg cells and inhibiting their differentiation into Th17 cells. This mechanism may also be mediated by alteration of the host intestinal microbiota. In conclusion, we provided evidence that the Th17/Treg imbalance is involved in the etiology of fibrosis in SSc and that enhancing Treg cells can ameliorate this imbalance and suppress the development of fibrosis. New therapeutic strategies for SSc fibrosis focusing on the regulation of the Th17/Treg imbalance are anticipated.

## Methods

### Patients

Thirty-six Japanese SSc patients 6 women and 10 men, mean age 63.7 ± 11.2 years (mean ± SD) and 20 age-, race-, and sex-matched healthy individuals were enrolled in this study. All SSc patients fulfilled the American College of Rheumatology/European League Against Rheumatism 2013 classification criteria^45^. Faecal samples were collected from all patients and healthy individuals for analysis of the intestinal microbiota. Seventeen patients were classified as limited cutaneous SSc (lcSSc) and 19 as diffuse cutaneous SSc (dcSSc) according to LeRoy et al.^46^. Skin sclerosis was assessed using the modified Rodnan skin thickness score^47^. Interstitial lung disease (ILD) was detected as bibasilar interstitial fibrosis or a ground-glass shadow visible on high-resolution computed tomography scans. Pulmonary artery hypertension (PAH) was defined as an elevated right ventricular systolic pressure by echocardiography, and subsequently, as an elevated mean pulmonary artery pressure by cardiac catheterization. Gastroesophageal reflux disease (GERD) was determined by endoscopic examination and/or symptoms of GERD. The study was approved by the institutional review board and the local research ethics committee of Gunma University (IRB2019-037 (1734)). All patients and volunteers were adults and provided written, informed consent before participating in the study. This study was conducted according to the principles of the Declaration of Helsinki.

### Bleomycin-induced skin fibrosis model

Dermal fibrosis was induced in 8-week-old C57BL/6 mice with injections of bleomycin. Injections of 300 µl of bleomycin (Nippon Kayaku, Tokyo, Japan) at a concentration of 1 mg/ml were given five times per week for 2 weeks as previously described^21,48^. Injections of 300 µl of PBS were used as controls for treatment with bleomycin. To examine the effect of Hp infection, mice were orally infected 200 L3 larvae 2 weeks before injections of bleomycin. The C57BL/6 mice were purchased from the SLC (Shizuoka, Japan). The mice were maintained in the Institute of Experimental Animal Research of Gunma University under specific pathogen-free conditions. At the end of each experiment, the mice were euthanized using carbon dioxide. All animal studies were approved by Gunma University Animal Care and Experimentation Committee. All methods used in animal experiments were conducted in accordance with relevant guidelines. The experimental procedures and reporting of this study were conducted in accordance with the ARRIVE guidelines (http://arriveguidelines.org).

### Helminth infection

Hp was maintained by serial passages of live mice. For experimental infections, faeces containing eggs from infected mice was incubated on filter paper soaked in distilled water, and the eggs were allowed to hatch and develop into infectious L3 larvae. Mice were orally infected with 200 L3 larvae as described previously^19^. Infection was monitored by detecting eggs in the faeces.

### Histological examination and immunofluorescence staining

Four-µm-thick sections of mice skin tissue embedded in paraffin were stained with hematoxylin and eosin (H&E) or Masson-Trichrome. Skin fibrosis was quantified using ImageJ software (version1.8.0, National Institutes of Health, Bethesda, MD) by measuring dermal thickness, which was defined as the distance from the epidermal-dermal junction to the dermal-subcutaneous junction, in 6 randomly selected microscopic fields. For immunohistochemical staining, tissue sections of mice skin were treated for antigen retrieval with a pressure cooker for 10 min at 121 °C. After blocking using Peroxidase Blocking (Dako, Glostrup, Denmark) for 5 min and Protein Block (Dako) for 10 min, the sections were incubated with anti-αSMA antibody (Sigma-Aldrich, St. Louis, MO), anti-CD3 antibody (Abcam, Cambridge, UK) and anti-CD68 antibody (Bio-Rad, Hercules, CA). After washing, the sections were incubated with a horseradish peroxidase-labeled polymer-conjugated secondary antibody (ENVISION+; Dako). Finally, color was developed with 3,3′-diaminobenzidine tetrahydrochloride. The positive cells for each staining were evaluated by counting the numbers of cells in 6 randomly selected microscopic fields.

### Quantitative assessment of collagen content

Total soluble collagen in the skin was quantified using a Sircol collagen assay (Biocolor, County Antrim, UK) according to the manufacturer’s protocol and the previously described protocols^21^.

### In vivo cell depletion

To deplete CD25-expressing cells including CD4^+^ Treg cells or CD122-expressing cells including CD8^+^ Treg cells in vivo, mice were injected intraperitoneally with 500 µg of anti-CD25 antibody (7D4) or anti-CD122 antibody (TMβ-1), respectively, or PBS as control, 1 day before and 3 and 5 days after the initiation of bleomycin injection. Both antibodies were kindly provided by H. Hisaeda (Department of parasitology, National Institute of Infectious Diseases, Tokyo, Japan).

### Flow cytometry

Single-cell suspensions from mouse inguinal lymph nodes were incubated with an anti-CD16/32 (93; eBioscience) to block Fc receptors. Cells were stained with the following monoclonal antibodies conjugated to PerCP-Cyanine5, allophycocyanin (APC) or phycoerythrin (PE) (TONBO biosciences or BioLegend): anti-mouse CD4 (GK1.5), anti-mouse CD25 (PC61.5), and anti-mouse IL-17RB (9B10). For intracellular staining, cells stained as described above were fixed and permeabilized with BD Cytofix/Perm (BD Bioscience) and then stained with anti-mouse Foxp3 (3G3) antibody. All fluorescent antibodies were used at 1/50 dilution. Stained cells were flowed through the FACSverse (BD Bioscience) and data were acquired using FACSDiva (BD Bioscience). Data analysis was performed using FlowJo 9.1 software (TreeStar).

### Intestinal microbiota analysis by 16 S rRNA sequencing

Faecal DNA extraction was performed according to a previous study with minor modifications^49^. A grain of mouse or human faeces pellets were suspended in TE10 buffer containing 10 mM Tris-HCl (pH 8.0) and 10 mM EDTA. The faecal suspension was incubated with 15 mg/ml lysozyme (Wako) at 37 °C for 1 h. A final concentration of 2000 units/ml of purified achromopeptidase (Wako) was added and then incubated at 37 °C for 30 min. 1% (wt/vol) sodium dodecyl sulfate and 1 mg/ml proteinase K (Merck Japan, Tokyo, Japan) were added to the suspension and incubated it at 55 °C for 1 h. After centrifugation, bacterial DNA was purified using phenol/chloroform/isoamyl alcohol (25:24:1) solution. DNA was precipitated by adding ethanol and sodium acetate. RNase A (Wako) was added to bacterial DNA in TE buffer to a final concentration 1 mg/ml. To remove fragmented low-molecular DNA, polyethylene glycol (PEG 6000) precipitation was performed after RNase treatment.

The V4 variable region (515 F–806R) was sequenced on an Illumina MiSeq, following the method of Kozich et al.^50^. Each reaction mixture contained 15 pmol of each primer, 0.2 mM deoxyribonucleoside triphosphates, 5 µl of 10× Ex Taq HS buffer, 1.25 U Ex Taq HS polymerase (Takara Bio), 50 ng extracted DNA, and sterilized water to a final volume of 50 µl. PCR conditions were as follows: 95 °C for 2 min, 25 cycles of 95 °C for 20 s, 55 °C for 15 s, and 72 °C for 5 min, followed by 72 °C for 10 min. The PCR product was purified by AMPure XP (Beckman Coulter, Brea, CA, USA). Real-time PCR for quantification was performed on a pooled library using a KAPA Library Quantification Kit for Illumina following the manufacturer’s protocols. A sample library with 20% denatured PhiX spike-in was sequenced by MiSeq using a 500-cycle kit. Taxonomic assignments and estimation of relative abundance from sequencing data were performed using the analysis pipeline of the QIIME2 version 2023.5^51^. Amplicon sequence variants (ASVs) were inferred from the denoised reads using DADA2^52^ implemented in QIIME2. The ASV taxonomy was assigned based on a comparison with the SILVA version 138^53^. β-Diversity was calculated using Bray-Curtis distances, which assess the dissimilarity in microbial community composition based on the OTU distribution across samples. Hierarchical clustering was performed to group the samples based on their microbial community similarities. The resulting Bray-Curtis distances were then visualized using Principal Coordinate Analysis (PCoA).

### Statistical analysis

*P*-values were calculated using Student’s t-test (two-tailed) or Chi-square analysis for comparison between two groups, and using one-way ANOVA followed by Tukey-Kramer test for the analysis of multiple groups. Data analysis was performed using GraphPad, version 9.4.1 (GraphPad Software, San Diego, CA). Error bars represent standard errors of the mean (SEM), and the numbers of experiments (n) are as indicated.

## Data Availability

Sequence data are available at the DNA Data Bank of Japan (DDBJ) with the accession codes DRA013870 (mice) and DRA13871 (SSc patients). Any additional data and materials regarding the study are available on request from the corresponding author.
